# ATP directly modulates thick filament structure and function in porcine myocardium

**DOI:** 10.1016/j.bpj.2025.06.037

**Published:** 2025-06-28

**Authors:** Marcus Rhodehamel, Meihua Guo, Vivek P. Jani, Hailey Flannagan, Shengyao Yuan, Maicon Landim-Vieira, Weikang Ma

**Affiliations:** 1 Department of Biomedical Engineering, The Johns Hopkins School of Medicine, Baltimore, MD; 2 Division of Cardiology, Department of Medicine, Johns Hopkins University School of Medicine, Baltimore, MD; 3 Institute for Genome Engineered Animal Models of Human Diseases, National Center of Genetically Engineered Animal Models for International Research, Dalian Medical University, Dalian, Liaoning, China; 4 BioCAT, Department of Biology, Illinois Institute of Technology, Chicago, Illinois; 5 Center for Synchrotron Radiation Research and Instrumentation, Illinois Institute of Technology, Chicago, Illinois

## Abstract

Cardiac contraction is achieved through cyclic cross-bridge interactions between overlapping myosin-containing thick filaments and actin-containing thin filaments. This process is powered by ATP hydrolysis by myosin, which must be sufficient for maintaining cardiac output. Myocardial ATP concentration is tightly maintained via several mechanisms. However, in decompensated end-stage heart failure, these mechanisms fail, resulting in depressed myocardial ATP levels, impaired cross-bridge kinetics, and reduced cardiac output. Here, we tested the hypothesis that ATP has a direct effect on thick filament activation by subjecting permeabilized porcine myocardium to increasing concentrations of ATP. Small-angle x-ray diffraction showed that higher ATP concentrations caused a structural transition in myosin heads from quasihelically ordered OFF states, where they are held in close proximity to the thick filament backbone, to disordered ON states, where they are free to move closer to thin filaments. Mechanically, high ATP did not alter maximum calcium-activated tension, although increasing ATP right-shifted the tension versus calcium (Ca^2+^) curve and accelerated both myosin attachment and detachment rates, consistent with prior studies. Power output and maximum unloaded shortening velocity also significantly increased with increased ATP concentration. Together, our structural and functional results indicate that ATP can directly turn thick filaments ON in porcine myocardium, suggesting a potential mechanism for the excessive proportion of myosin in the inactivated state in certain heart diseases. The profound effect on cross-bridge kinetics also suggests that reduced ATP concentration impairs relaxation and may also play a role in diastolic dysfunction.

## Significance

The heart requires a daily energy expenditure of 6–35 kg of ATP, which is orders of magnitude higher than the myocardial ATP pool and, as such, ATP concentration is tightly controlled by multiple mechanisms in the heart. Our x-ray diffraction results showed that ATP can directly turn thick filaments ON that lead to altered cross-bridge kinetics affecting muscle activation and relaxation. Our study suggests that depressed cellular ATP levels might, at least partially, contribute to the excessive proportion of myosin in the inactivated state observed in certain heart diseases.

## Introduction

Cardiac contractility and contractile reserve are inherently linked to cardiac metabolism as the chemical energy generated from ATP hydrolysis is critical for actin-myosin cross-bridge cycling. Approximately 60–70% of the ATP generated in the cardiomyocyte is used for interactions between myosin-containing thick filaments and actin-containing thin filaments, with the remaining used by calcium handling proteins (1). Sufficient ATP reserves are critical for ensuring adequate cardiac output and sufficient end-organ perfusion. The heart requires a daily energy expenditure of 6–35 kg of ATP (2), orders of magnitude higher than the myocardial ATP pool. The ATP reserve in cardiomyocytes, however, is only sufficient to support heart beats for a few seconds (3). It is therefore crucial to maintain high efficiency in energy production, as even subtle changes in ATP generation or utilization can profoundly impact cardiac function (1,3,4,5,6). Reduction in intracellular ATP concentration ([ATP]) has been commonly observed in various heart diseases (7,8,9). In these diseases, several studies have identified reduced myocardial [ATP], which declines from ∼8 mM as seen in the healthy heart to 2–4 mM, seen in the failing heart (10,11,12,13,14,15,16). This reduction in intracellular [ATP] has led to the hypothesis that diminished [ATP] directly contributes to diastolic dysfunction, inadequate perfusion, and mortality in heart failure. Previous studies that showed that actomyosin ATPase activity in vitro (17,18), in skeletal (19), and cardiac tissue (4) are [ATP] independent at millimolar [ATP] suggest that the reduced [ATP] observed in heart failure may not affect myocardium contraction appreciably. Beard et al. (20), however, showed that reduction in [ATP] between 2 and 8 mM reduces maximal power output and slows cross-bridge cycling kinetics in rodent cardiac tissue suggesting a direct effect of [ATP] to cross-bridge kinetics, but details of this mechanism are lacking.

The aim of this study is to investigate the effects of [ATP] on the structure and function of porcine myocardium. Structurally, our findings demonstrate that higher [ATP] induces a transition in myosin heads from quasihelically ordered “OFF” states, where they are held close to the thick filament backbone, to disordered “ON” states, where they move closer to the thin filaments. Note that the ON state, as defined here, is a relaxed state that is distinct from the actin-activated state when cross-bridges are formed and force is produced. Mechanically, elevated [ATP] did not alter maximum calcium-activated tension, but did shift the steady-state tension-calcium relationship to the right and accelerated both myosin attachment and detachment rates. Additionally, power output and maximum unloaded shortening velocity significantly increased with increased [ATP]. These results demonstrate that ATP can directly activate thick filaments in porcine myocardium, providing a plausible link between metabolism and the degree of thick filament activation. These observations suggest a possible mechanism for the excessive degree of myosin inactivation and impaired relaxation seen in certain cardiac and skeletal myopathies.

## Materials and methods

### Ethical approval

Previously frozen wild-type porcine hearts (biological repeats *N* = 2) were purchased from Exemplar Genetics. The tissues were obtained in compliance with protocols approved by the Institutional Animal Care and Use Committees of Exemplar Genetics and no live animals were directly involved in this study. As a result, no institutional approval was required by the Illinois Institute of Technology and Johns Hopkins University.

### Muscle preparation

Porcine left ventricular myocardium samples were prepared following the methodology outlined in our previous study (21). Briefly, frozen muscles were thawed and permeabilized in cardiac relaxing solution (2.25 mM Na_2_ATP, 3.56 mM MgCl_2_, 7 mM EGTA, 15 mM sodium phosphocreatine, 91.2 mM potassium propionate, 20 mM imidazole, 0.165 mM CaCl_2_, 15 U/mL creatine phosphate kinase, and protease inhibitor cocktail [Roche, Switzerland (Base)]) with added 15 mM 2,3-butanedione 2-monoxime (BDM) and 1% Triton X-100 for ∼30 min before splitting them into smaller fiber bundles (∼500 *μ*m in diameter and ∼5 mm long). These fiber bundles were then transferred to fresh skinning solution and left to incubate overnight at 4°C on a rocker. Subsequently, the muscles underwent three washes with fresh cardiac relaxing solution, each lasting 10 min, to wash out the BDM and Triton X-100. The muscle bundles were further dissected into strips with a diameter of approximately 200 *μ*m and clipped with aluminum T-clips and stored in cold relaxing solution (2.25 mM Na_2_ATP, 3.56 mM MgCl_2_, 7 mM EGTA, 15 mM sodium phosphocreatine, 91.2 mM potassium propionate, 20 mM imidazole, 0.165 mM CaCl_2_) containing 3% dextran at 4°C for use in experiments on the same day. For high ATP solution (7.25 mM), the MgCl_2_ concentration was increased proportionally to maintain a constant free magnesium concentration while potassium propionate concentration was reduced to maintain the ionic strength (7.25 mM Na_2_ATP, 12 mM MgCl_2_, 7 mM EGTA, 15 mM sodium phosphocreatine, 20.9 mM potassium propionate, 20 mM imidazole, 0.165 mM CaCl_2_). The intermediate level of ATP (4.75 mM) solution was achieved by mixing the high (7.25 mM) and low (2.25 mM) relaxing solution equally.

### X-ray diffraction

X-ray diffraction experiments were performed at the BioCAT beamline 18ID at the Advanced Photon Source, Argonne National Laboratory (22). The x-ray beam energy was set to 12 keV (0.1033 nm wavelength) at an attenuated incident flux of ∼5 × 10^12^ photons per second (∼50% of the full beam). The specimen-to-detector distance was ∼3 m. The preparation was then attached at one end to a hook on a force transducer (Model 402B Aurora Scientific, Aurora, ON, Canada) and the other end to a static hook. The muscle was incubated in a custom chamber attached to a heat exchanger so that the solution was maintained between 28 and 30°C. The muscles were stretched to a sarcomere length of 2.0 *μ*m by adjusting the manipulator attached to the force transducer while monitoring the light diffraction patterns from a helium-neon laser (633 nm). X-ray patterns were first collected in relaxing solution with 2.25 mM ATP and then in solutions with elevated ATP (4.75 and 7.25 mM, respectively) with 3% dextran throughout using a MarCCD 165 detector (Rayonix, Evanston, IL) with a 1 s exposure time.

The muscle samples were oscillated along their horizontal axes at a velocity of 1–2 mm/s to minimize radiation damage. The irradiated areas are moved vertically after each exposure to avoid overlapping x-ray exposures. Two or three patterns were collected under each condition.

### X-ray diffraction data analysis

The data were analyzed using the MuscleX software package (23). The equatorial reflections were measured using the “Equator” routine in MuscleX as described previously (24). For subsequent analyses, the x-ray patterns were quadrant folded and background subtracted using a circularly symmetric background model implemented in the “Quadrant Folding” routine in MuscleX. The intensities and spacings of meridional and layer line reflections were measured by the “Projection Traces” routine in MuscleX as described previously (25). Briefly, the spacing of the peak was estimated as the centroid of the intensity of the top half of the diffraction peak (26) and the integrated intensity under a diffraction peak is modeled as the area of a Gaussian function (23). To compare the intensities under different conditions, and correct for any possible x-ray diffraction intensity changes due to radiation damage to the muscle, the measured intensities of x-ray reflections are normalized to the sixth-order actin-based layer line intensities as described previously (27). There were no significant changes in the radial width of the M3 reflection among different conditions, thus no correction for width was applied to the measured intensities. Values obtained from patterns under the same condition were averaged.

### Isometric tension-calcium relationships

Tension-calcium concentration ([Ca^2+^]) curves were obtained from skinned cardiomyocytes, as described previously (28,29,30,31,32). Myocytes were homogenized in skinning solution (5.55 mM Na_2_ATP, 7.11 M MgCl_2_, 2 mM EGTA, 108.01 mM KCl, 8.91 KOH, 10 mM imidazol, 10 mM DTT + 0.3% Triton X-100) with protease (Sigma-Aldrich, MO) and phosphatase inhibitors (PhosSTOP, Roche, Germany) and skinned for 20 min at 4°C. Myocytes were attached to a force and length controller (Force Transducer: 403A, Length Controller: 315C, Digital Controller; 600A, Aurora Scientific, Canada) using an ultraviolet-activated adhesive (Norland Optical Adhesive 63, Norland, NJ), moved into room temperature (∼22°C) relaxing buffer (5.95 mM MgATP, 6.41 mM MgCl_2_, 10 mM EGTA, 100 mM BES, 10 mM CrP, 50.25 mM K propionate, protease inhibitor [Sigma-Aldrich, St. Louis, MO], 1 mM DTT). All tension-[Ca^2+^] curves were acquired at the sarcomere length of 2.1 *μ*m, measured by 1D fast Fourier transform (Aurora Scientific Software, IPX-VGA210, Imperx, FL).

Tension-Ca^2+^ relationships were acquired by varying Ca^2+^ concentration from 0.0 to 46.8 μM. Force was normalized to the cross-sectional area estimated as $\frac{\pi }{4}ab$, where *a* is the diameter of the myocyte from the camera and *b* is the short axis diameter approximated as 0.8*a*, to obtain tension (mN/mm^2^). Cardiomyocytes were subsequently moved to relaxing buffer with either 2- or 8-mM ATP and the experiments repeated. The steady-state tension versus log[Ca^2+^] plots (T-Ca^2+^ plots) were fit to the three-element Hill equation: T = T_max_ × Ca^*nh*^/(EC_50_^*nh*^ + Ca^*nh*^), where T_max_ is maximum calcium-activated tension, EC_50_ is calcium sensitivity, and n_h_ is the Hill coefficient. Calcium concentrations that are reported are free calcium, calculated as previously described (33,34), using the MaxChelator program.

### Cross-bridge kinetics

Cross-bridge kinetics, parameterized as 2πb and 2πc, were acquired using a step response as described previously (35,36). Cardiomyocytes were prepared as described above. The cardiomyocyte was set to a sarcomere length of 2.1 *μ*m. The cardiomyocyte was then moved to saturating calcium concentrations ([Ca^2+^] = 46.8 *μ*M). After steady-state force was achieved, a stepwise lengthening (2% cardiomyocyte length [CL], <0.15% sarcomere length) was applied, and force measured for 7 s at 2000 Hz. The resulting force trace was normalized such that $\bar{F}$ = (F − F_max_)/F_step_, where $\bar{F}$ is the normalized force, F_max_ is the steady-state force, and F_step_ is the force immediately after the step response. The force trace was then fit to the equation $\bar{F}\left(t\right)=P_{1}+P_{2}e^{-2\pi bt}-P_{3}e^{-2\pi ct}$, from which myosin attachment (2πb) and detachment (2πc) rates were acquired. The experiments were subsequently repeated at 2 and 8 mM ATP.

### Velocity and power in cardiomyocyte contraction

Tension-velocity and tension-power relationships were obtained using a force clamp protocol as described previously (31). Cardiomyocytes were set to SL 2.1 *μ*m and subjected to 3.8 *μ*M Ca^2+^ and were allowed to isometrically contract to steady state. After steady-state force was achieved, cardiomyocytes were subjected to a fixed load and allowed to shorten. Cardiomyocytes were first subjected to loads of ∼90% maximum steady-state force (F_max_). Force was decremented in 5–10% in a stepwise fashion, and the CL-time trace was obtained for each load. Velocity of cardiomyocyte shortening was measured for at least 75 ms immediately following the change in tension using linear regression of the length-time tracing to account for internal viscous load in cases of an exponential length-time trace. Force was directly measured from the force transducer for all measurements to correct for potential errors in servo control. Measured velocity was normalized to CL for each cardiomyocyte. Power was calculated as the product of tension (mN/mm^2^) and velocity normalized to CL to obtain units W/m^3^. Data were fit to the hyperbolic Hill equation, (T + a) (V + b) = (T_max_ + a)b to determine V_max_ (CL/s) and P = Tb((T_max_ + a)/(T + a) − 1) and peak power P_max_ (mW/cm^3^). Data were fit using custom protocols in MATLAB (The MathWorks, Natick, MA, 2024b).

### Statistical analyses

**Figure 1:**
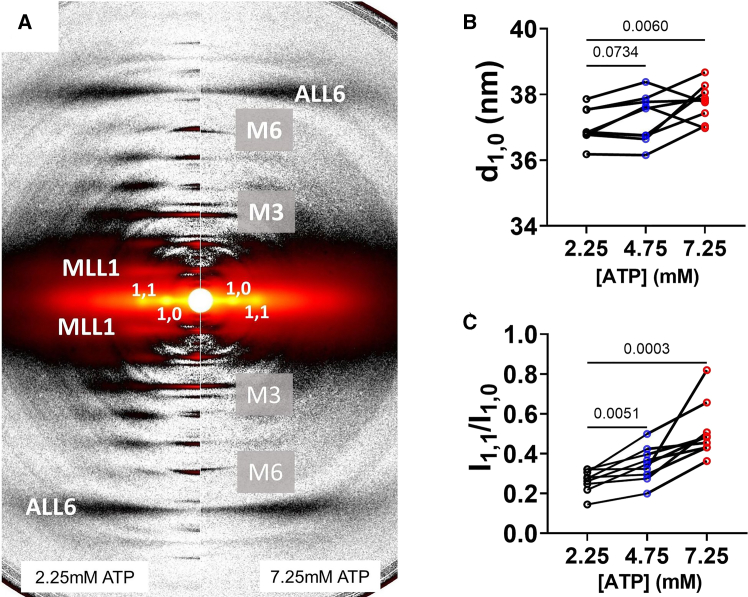
Effects of ATP on x-ray diffraction patterns. (*A*) Representative x-ray diffraction patterns from permeabilized relaxed pig myocardium at low (2.25 mM, *left panel*) and high (7.25 mM, *right panel*) concentrations of ATP. (*B*) Lattice spacing (d_1,0_) from permeabilized pig myocardium at different [ATP]. (*C*) Equatorial intensity ratio (I_1,1_/I_1,0_) from permeabilized pig myocardium at different [ATP]. *N* = 2 (biological replicates) and *n* = 9 (technical replicates).

**Figure 2:**
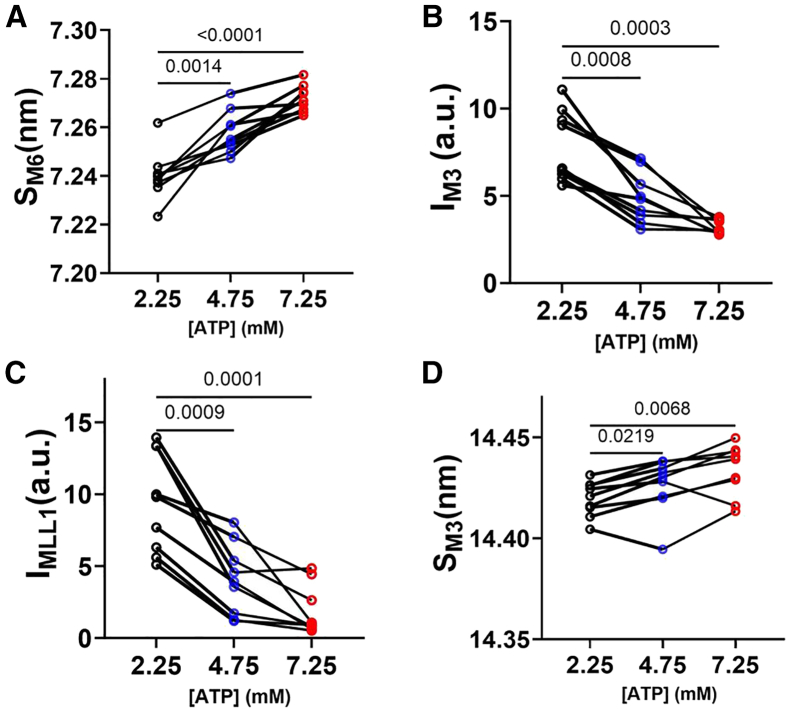
Effects of ATP concentration on myosin layer lines and meridional reflections on permeabilized pig myocardium. (*A*) Spacing of the sixth-order myosin meridional (S_M6_) at different ATP levels. (*B*) Intensity of the third-order myosin meridional (I_M3_) at different ATP levels. (*C*) Intensity of the first-order myosin-based layer lines (I_MLL1_) at different ATP levels. (*D*) Spacing of the third-order myosin meridional (S_M3_) at different ATP levels (*N* = 2; *n* = 9).

**Figure 3:**
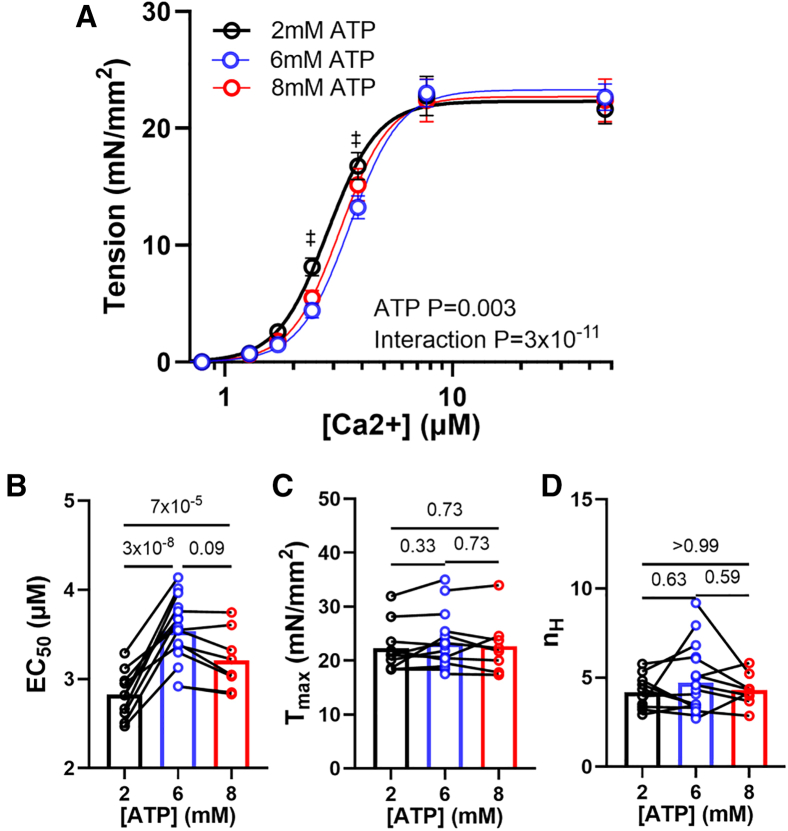
Static cardiomyocyte mechanics from permeabilized porcine myocardium at different [ATP]. (*A*) Active tension in a function of calcium concentration in solutions with different [ATP]. (*B*) Calcium concentration to achieve half-maximal activation (EC_50_), (*C*) Maximum tension generated under saturating calcium conditions (T_max_), and (*D*) Hill coefficient (n_H_) of the tension calcium relationship in solutions with different [ATP] (*N* = 2; *n* = 8–18). Error bars presented as the standard error of the mean (SEM).

**Figure 4:**
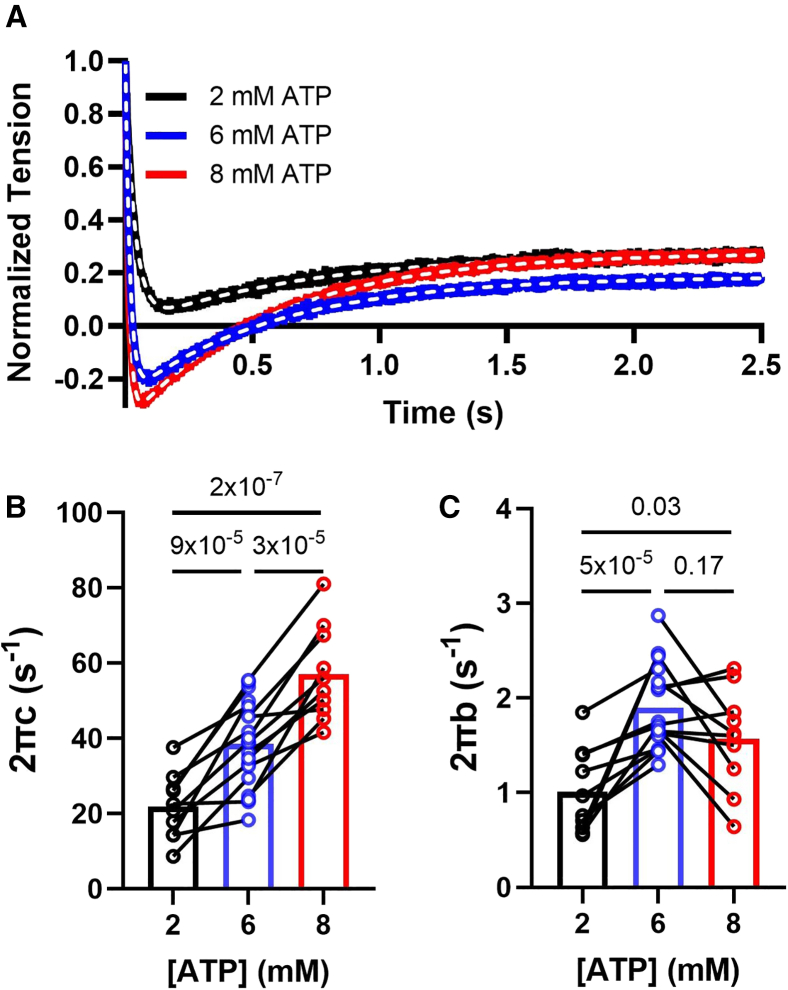
Cross-bridge kinetics from permeabilized porcine cardiomyocytes at different [ATP]. (*A*) Representative normalized force traces and their corresponding fits (*white dashed lines*) from cardiomyocyte after a 2% stepwise lengthening at different [ATP]. The cross-bridge detachment rate (2πc, *B*) and cross-bridge attachment rate (2πb, *C*) from cardiomyocytes at different [ATP] (*N* = 2; *n* = 10–20).

**Figure 5:**
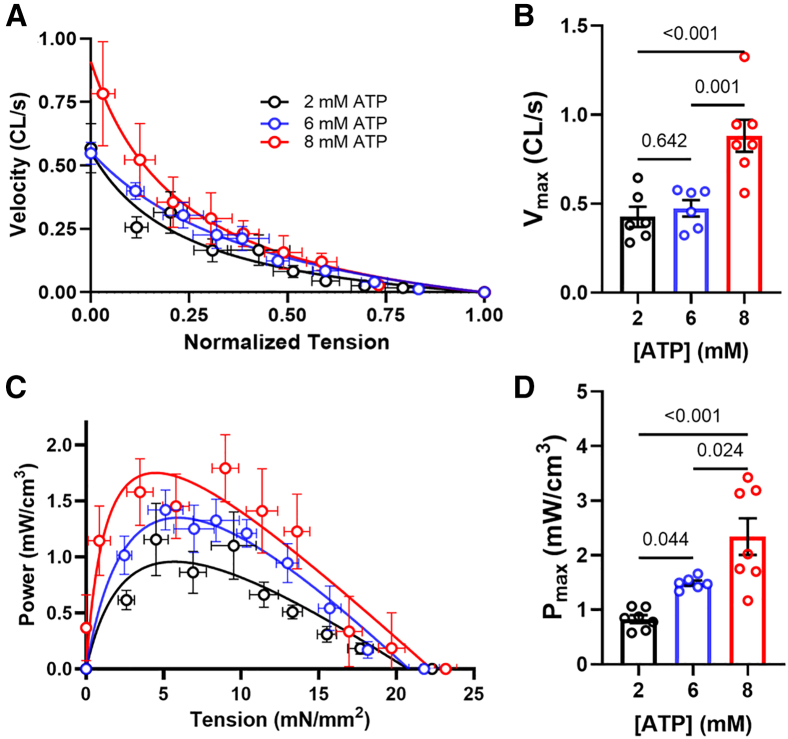
Tension, velocity, and power relationships from permeabilized porcine cardiomyocytes at different [ATP]. (*A*) Tension-velocity relationship of porcine cardiomyocytes at different [ATP] (CL, cardiomyocyte length). (*B*) Maximum shorting velocity of porcine cardiomyocytes at different [ATP]. (*C*) Tension-power relationship of porcine cardiomyocytes at different [ATP]. (*D*) Maximum power output of porcine cardiomyocytes at different [ATP] (*N* = 2; *n* = 6–9). Error bars presented as the standard error of the mean (SEM).

Statistical analyses were performed using GraphPad Prism 10.0.2. Results in the text and error bars in the figures are given as mean ± standard error of mean. Paired one-way ANOVA with Geisser-Greenhouse correction and multiple comparisons were used in Figs. 1 and 2; mixed-effects models with assumed sphericity with multiple comparisons were used in Figs. 3 and 4, and ordinary one-way ANOVA with Holm-Sidak’s multiple comparisons were used in Fig. 5 to compare values obtained from samples incubated in different ATP concentrations.

## Results

We investigated structural changes in permeabilized porcine myocardium as the [ATP] in relaxing solution increase. Increasing ATP concentration in relaxing solution weakens the overall intensities of the myosin-based reflections, notably the third-order myosin meridional reflection (M3) and the first myosin layer line (MLL1) (Fig. 1 *A*). The equatorial reflections arise from the hexagonally packed myofilaments inside the sarcomere, while the meridional reflections originate from the axial repeating structures of myofilaments (37). The interfilament lattice spacing, d_1,0_, directly proportional to the thick-to-thick filament spacing, increased from 37.16 ± 0.17 nm at 2.25 mM ATP to 37.28 ± 0.24 and 37.86 ± 0.18 nm at 4.75 and 7.25 mM ATP, respectively (Fig. 1 *B*). The ratio of the intensity of the 1,1 equatorial reflection to that of the 1,0 equatorial reflection, I_1,1_/I_1,0_, is an indicator of the proximity of myosin heads to actin (24,38) in the relaxed state. I_1,1_/I_1,0_ increased from 0.26 ± 0.02 nm at 2.25 mM ATP to 0.34 ± 0.03 and 0.52 ± 0.05 nm at 4.75 and 7.25 mM ATP, respectively (Fig. 1 *C*). The increase of I_1,1_/I_1,0_ indicates that myosin heads shift radially away from the thick filament backbone toward the actin-containing thin filaments as ATP concentration increases in diastolic calcium level.

Under resting conditions, the majority of the myosin heads are quasihelically ordered on the surface of the thick filament, with higher diffraction intensity in the myosin layer lines indicating better ordering (39,40). These helically ordered myosin heads are structurally defined as OFF heads that are less likely to interact with actin. These OFF heads need to be turned ON to participate in contraction. An increase in thick filament backbone periodicity, as demonstrated by an increase in the M6 meridional reflection spacing (S_M6_), a reduction in the degree of the helical ordering of the myosin heads as demonstrated by a reduction of the intensity of the first-order myosin-based layer line (I_MLL1_) and the third-order myosin-based meridional reflection (I_M3_) are indicators of more myosin heads adopting ON states (37,41). S_M6_ increased from 7.240 ± 0.003 nm at 2.25 mM ATP to 7.258 ± 0.003 and 7.272 ± 0.002 nm at 4.75 and 7.25 mM ATP, respectively (Fig. 2 *A*). Furthermore, I_M3_ decreased from 7.81 ± 0.68 nm at 2.25 mM ATP to 4.90 ± 0.48 and 3.23 ± 0.14 nm at 4.75 and 7.25 mM ATP, respectively (Fig. 2 *B*). Simultaneously, I_MLL1_ decreased from 9.47 ± 1.17 nm at 2.25 mM ATP to 4.08 ± 0.82 and 1.87 ± 0.57 nm at 4.75 and 7.25 mM ATP, respectively (Fig. 2 *C*). The M3 meridional reflection spacing (S_M3_), which originates from the axial periodicity of the myosin heads, has been shown to be associated with the OFF/ON transition. In slow porcine ventricular myocardium, the structural ON state is characterized by a reduction of S_M3_ (42). S_M3_, however, increased significantly, albeit small, from 14.420 ± 0.003 nm at 2.25 mM ATP to 14.426 ± 0.005 and 14.434 ± 0.004 nm at 4.75 and 7.25 mM ATP, respectively (Fig. 2 *D*). These results suggest that ATP may induce a different OFF-to-ON transition of myosin compared with the calcium-induced transition observed in previous studies (42,43).

The contractile tension versus –log[Ca^2+^] curves from permeabilized porcine cardiomyocytes at different [ATP] showed a classical sigmoidal relationship and were subsequently fit to the Hill equation (Fig. 3 *A*). The addition of ATP induced a right-upward shift in the tension/–log[Ca^2+^] relationship as indicated by an increase in EC_50_, the concentration of calcium to achieve half-maximal activation and a measure of calcium sensitivity, from 2.82 ± 0.08 *μ*M at 2 mM of ATP to 3.54 ± 0.08 and 3.21 ± 0.12 *μ*M at 6 and 8 mM ATP, respectively, although there was no significant difference in EC_50_ between 6 and 8 mM ATP (Fig. 3 *B*). The right-upward shift in the tension/–log[Ca^2+^] relationship resulting a significant increase in tension at submaximal calcium concentrations (1.71 and 2.41 *μ*M, *p* < 0.05; Fig. 3 *A*) without significantly increasing the maximal tension (T_max_, 22.30 ± 1.42, 23.25 ± 1.14, and 22.70 ± 1.85 mN/mm^2^ at 2, 6, and 8 mM ATP, respectively; Fig. 3 *C*). No significant differences were observed in the Hill coefficient (4.19 ± 0.30, 4.72 ± 0.44, and 4.30 ± 0.32 at 2, 6, and 8 mM ATP, respectively; Fig. 3 *D*).

The cross-bridge cycling kinetics under different [ATP] was investigated by applying a 2% stepwise lengthening to the cardiomyocyte preparations to disrupt cross-bridge attachment and allow cross-bridge reattachment to determine the cross-bridge detachment rates (2πc) and attachment rates (2πb) during maximal activation. Both 2πc (21.91 ± 2.70, 38.54 ± 2.49, and 57.07 ± 3.93 s^−1^ at 2, 6, and 8 mM ATP, respectively; Fig. 4 *A*) and 2πb (1.01 ± 0.14, 1.90 ± 0.10, and 1.57 ± 0.17 s^−1^ at 2, 6, and 8 mM ATP, respectively; Fig. 4 *A*) values are significantly increased at higher [ATP] compared with at low [ATP] (2 mM) indicating an increased cross-bridge cycling rate in the presence of extra ATP, consistent with a prior report (20).

Cardiomyocytes were then subjected to isotonic loads and allowed to shorten to obtain maximum shortening velocity and maximum power production. Shortening velocities normalized to CL in cardiomyocytes at different [ATP] were plotted as a function of normalized tension (Fig. 5 *A*). The tension-velocity curve deviated toward higher velocities in the lower tension region for the cardiomyocytes subjected to 8 mM [ATP] compared with the cardiomyocytes subjected in lower [ATP] (2 and 6 mM). Quantitatively, the maximal shortening velocity (V_max_), estimated by extrapolating the tension-velocity relationships to zero force, were significantly higher for the cardiomyocytes subjected to 8 mM [ATP] (0.88 ± 0.09 CL/s) compared with the cardiomyocytes subjected in lower [ATP] (0.43 ± 0.06 and 0.47 ± 0.05 CL/s at 2 and 6 mM [ATP], respectively; Fig. 5 *B*). The power output of cardiomyocytes, calculated by multiplying tension by velocity, at different [ATP], were plotted as a function of absolute tension. The tension-power relationship shifted upwards as [ATP] increased (Fig. 5 *C*), consistent with a prior report (20). Quantitatively, maximal power (P_max_), estimated by fitting the tension-power curve to the power velocity Hill equation, increased significantly from 0.83 ± 0.07 mW/cm^3^ at 2 mM [ATP] to 1.49 ± 0.05 and 2.34 ± 0.34 mW/cm^3^ at 6 and 8 mM [ATP], respectively (Fig. 5 *D*).

## Discussion

### ATP directly activates thick filaments

Our small-angle x-ray diffraction results from porcine myocardium showed that myosin heads move from being in close proximity to the thick filament backbone toward the thin filament, as indicated by an increase in I_1,1_/I_1,0,_ as [ATP] increases. This shift is accompanied by a transition from ordered to disordered states, as indicated by the reduction of I_M3_ and I_MLL1_. These results demonstrate that ATP promotes the transition of myosin from the OFF state to the ON state, thereby directly activating the thick filament. ATP cycling, including rate of ATP hydrolysis, rate of ADP release, and ATP-induced actomyosin dissociation has been shown to be independent of ATP concentrations in the mM range based on actomyosin ATPase (17,18) as well as in rodent skeletal (19) and cardiac tissue (4). In contrast, one study (44) showed that cross-bridge detachment rate in porcine myocardium increases at high [ATP], in the range of 0.025–5 mM, suggesting that [ATP] might not be fully saturating, at least in this muscle. In the OFF state, myosin is thought to adopt a folded interacting head motif in the ADP + Pi state. The exchange of ADP and Pi for ATP requires the opening of the nucleotide-binding pocket within the globular head region of myosin. Once bound, newly bound ATP is hydrolyzed to ADP and Pi and has the potential to reenter the interacting head motif state. This cycle of nucleotide exchange requires significant structural changes of myosin heads that could disrupt the OFF state of myosin. In summary, our data suggests a mechanism whereby an increase in [ATP] directly activates the thick filament.

### [ATP] affects muscle mechanics

It has been proposed that the ensemble force (F_ens_) of muscle contraction can be expressed as F_ens_ = F_int_ × N_a_ × t_s_/t_c_, where F_int_ is the intrinsic force of a cross-bridge, t_s_/t_c_ is the duty ratio (the proportion of time myosin is bound to actin during the cross-bridge cycle), and N_a_ is the number of myosin heads available for force production (45). The concept of thick filament-based regulation involves altering N_a_, and a leftward shift in the tension versus Ca^2+^ curve is expected when the thick filament is activated, as shown with other myosin activators such as EMD (32), dATP (27), or danicamtiv (44).

In this study, we observed that, whereas high [ATP] activated the thick filament (Figs. 1 and 2), the mechanical tension versus Ca^2+^ curve shifted rightward without significantly affecting maximum calcium-activated tension (Fig. 3) consistent with previous studies on rat myocardium (20,46). Consistent with previous findings (20), maximum power output increased with elevated [ATP]. However, while we observed an increase in maximum unloaded shortening velocity, the previous study did not detect this change (20). High [ATP] has been shown to increase the cross-bridge detachment rate in porcine myocardium (44). This is supported by the observed increase in 2πc observed in cardiomyocytes with increasing [ATP]. We therefore hypothesize that accelerated myosin detachment likely explains the rightward shift in the tension versus Ca^2+^ curve and depressed isometric force production at intermediate calcium levels. The reduction in isometric tension at systolic calcium concentrations does not necessarily indicate a decrease in heart performance. Since the heart shortens during contraction, power output serves as a more relevant index of heart performance than isometric tension. The observation that [ATP] increases power may explain previous findings that increasing [ATP] enhances cardiac output (14). Notably, the force-velocity relationship modeled by Beard et al. did not reproduce the ATP-dependent changes in power at equivalent ATP concentrations (20). The exact reason for the model’s failure to recapitulate the experimental results is unknown; we believe, however, that further improvements to the model may be necessary to resolve this issue.

### Implications for experiments on relaxed muscle

ATP is required for myosin to detach from actin during the cross-bridge cycle. Therefore, ATP, typically around 5–6 mM, is added to solutions when preparing muscle samples for in vitro and in situ physiological experiments. Adequate [ATP] is essential to prevent muscle from entering a state of rigor, keeping it relaxed from a mechanical standpoint. Our x-ray studies, however, suggest that myosin heads at the presumptive relaxed [ATP] (∼5 mM) are not necessarily structurally relaxed in permeabilized porcine myocardium. It has been generally assumed that the majority of myosin heads adopt quasihelical ordered states when relaxed, which produce the characteristic myosin-based meridional and layer line reflections. However, recent cryo-EM and TEM studies have shown that the majority of myosin heads i.e., one-third of heads in the C zone and all heads in the D zone, are disordered even in the presence of the myosin inhibitor mavacamten (47,48,49).

It was surprising to observe that I_MLl1_ dropped by ∼57 and 80% under these same conditions (Fig. 2 *D*). Assuming that the diffracted intensity is proportional to the square of the total electron density (mass), we estimate that the population of myosin heads in the quasihelically ordered state decreased by approximately 25 and 55% as [ATP] increased from 2.25 to 4.75 mM and 7.25 mM, respectively. The relatively high resolution of the cardiac thick filament structures obtained by Tamborrini et al. (49) and Dutta et al. (48) were achieved by relaxing the muscle at ∼ 5 mM ATP in the presence of mavacamten. It is reasonable to speculate that more myosin on the cardiac thick filament would adopt the OFF structural state and improve helical ordering at even lower ATP concentrations (<2.25 mM). Notably, a recent study estimated that the diastolic [ATP] in mouse cardiomyocytes is less than 1 mM, approximately one order of magnitude lower than previously reported (50). From a structural perspective, this finding supports the general assumption that the majority of myosin heads adopt quasihelical ordered states when relaxed in vivo. While this is beyond the scope of the current study, determining the optimal conditions, such as ATP concentration, ionic strength, and other factors, for preserving the integrity of thick filament structure in situ would be highly valuable.

### Implications of [ATP] in heart disease

Numerous studies have consistently identified reduced intracellular [ATP] is a key characteristic of advanced and end-stage heart failure (5,8,20). Consequently, enhancing cardiac energetics has long been considered a promising therapeutic approach (2,5) and many early studies have shown that enhancing myocardial ATP production pharmacologically has had benefit in patients with acute ischemia (51). More recently, it has been observed that the only effective and clinically indicated treatments for heart failure with preserved ejection fraction (HFpEF), which now accounts for more than 50% of all heart failure cases globally, were initially developed for diabetes and are hypothesized to increase myocardial contraction and relaxation and improve outcomes by enhancing cardiac metabolism (52,53,54,55).

Depression of myocardial contractility is commonly seen in heart failure independent of ejection fraction (28,56,57,58,59,60). The underlying mechanisms contributing to depressed myocardial contractility in heart failure, however, remain an area of ongoing investigation. Our recent study suggests that thick filament inactivation may be a mechanism (31) underlying depressed myocardial contractility in heart failure. However, the mechanisms responsible for this myosin inactivation are still unclear. The observation that [ATP] has a profound structural and mechanical effect on the thick filament leads to the intriguing hypothesis that the depressed intracellular [ATP] in heart failure might, at least partially, contribute to the myosin inactivation seen in these patients. Additionally, while this study focuses on the effect of [ATP] in healthy tissue, the disparity in intracellular [ATP] between healthy and diseased myocardium raises the question of whether myocardial contractility, typically assessed in situ under uniform [ATP] conditions, accurately reflects the pathological conditions present in a diseased heart.

Given that ATP is the primary energy source for muscle contraction, it is reasonable to expect that [ATP] can affect myocardial function. The pool of [ATP] available to myosin for the formation of cross-bridges has been a limiting factor for therapies that augment myocardial contractility. Myotropic contractile stimulants (e.g., omecamtiv mecarbil, danicamtiv, and others) are attractive because they do not have the same negative effect on cardiac energetics that inotropes do (61), even though these agents have only yielded modest improvements in clinical outcomes (62). Given that ATP concentration is reduced in heart failure along with the structural and mechanical effects identified here, it is possible that the reduced efficacy of these therapies in vivo may be a consequence of reduced thick filament activation and slowed cross-bridge kinetics from low ATP in heart failure. Future studies are required to identify whether this is indeed the case.2

### Study limitations

While our mechanical assays were conducted with matched magnesium concentration through the use of MgATP, the ionic strength was not matched. Although our mechanical results generally align with the findings of Beard et al. (20), it is possible that the increased ionic strength due to the additional ATP contributed to the mechanical properties observed here. Furthermore, while we speculate that altered ATP levels might contribute to myosin inactivation observed in certain heart diseases, our study did not directly investigate disease states. Additionally, although ATP production and consumption are central to many aspects of cardiac metabolism (3,63), metabolic pathways are complex, and metabolites from both ATP-producing and non-ATP-producing pathways play critical roles in regulating cardiomyocyte function. Therefore, further studies are required to establish a direct link between reduced ATP levels and cardiac dysfunction in heart disease.

## Data and code availability

The data sets generated or analyzed during this study are included in this article. The raw data are available from the corresponding authors, Vivek Jani (vjani1@jhmi.edu) and Weikang Ma (wma6@iit.edu) on reasonable request.

X-ray data sets were analyzed using data reduction programs belonging to the open-source MuscleX software package developed at BioCAT. MuscleX source codes: https://github.com/biocatiit/musclex.

## Acknowledgments

We thank Dr. Saffie Mohran of the University of Washington for his assistance with the x-ray experiments. This project is supported by 10.13039/100000050National Heart, Lung, and Blood Institute grant (R01HL171657, to W.M.), National Institute of Health predoctoral fellowship grant (F31 HL168850, to V.P.J.), and an 10.13039/100000968American Heart Association predoctoral fellowship grant (23PRE1026275, to V.P.J.). This research used resources of the Advanced Photon Source, a U.S. Department of Energy (DOE) Office of Science User Facility operated for the DOE Office of Science by 10.13039/100006224Argonne National Laboratory under contract no. DE-AC02-06CH11357. BioCAT is supported by grant P30 GM138395 from the 10.13039/100000057National Institute of General Medical Sciences of the 10.13039/100000002National Institutes of Health. The content is solely the authors' responsibility and does not necessarily reflect the official views of the National Institute of General Medical Sciences or the National Institutes of Health.

## Author contributions

V.P.J. and W.M. designed the experiments. M.R., V.P.J., H.F., S.Y., M.L.-V., and W.M. performed the experiments. M.R., V.P.J., M.G., and W.M. analyzed the data. V.P.J. and W.M. wrote the manuscript. All authors approved the final version of the manuscript.

## Declaration of interests

W.M. consults for Edgewise Therapeutics, Cytokinetics Inc. and Kardigan Bio, but this activity has no relation to this work.
